# The Relation of Body Mass Index to Muscular Viscoelastic Properties in Normal and Overweight Individuals

**DOI:** 10.3390/medicina57101022

**Published:** 2021-09-26

**Authors:** Serkan Usgu, Engin Ramazanoğlu, Yavuz Yakut

**Affiliations:** 1 Physiotherapy and Rehabilitation Department, Faculty of Health Science, Hasan Kalyoncu University, 27010 Gaziantep, Turkey; yyakut@yahoo.com; 2 Physiotherapy and Rehabilitation Department, Faculty of Health Sciences, Inonu University, 44280 Malatya, Turkey; enginramazanoglu@hotmail.com

**Keywords:** body mass index, tone, stiffness, elasticity, overweight

## Abstract

*Background:* The body mass index (BMI) is closely related to fat tissue, which may have direct or indirect effects on muscle function. Previous studies have evaluated BMI and muscle viscoelastic properties in vivo in older people or individual sexes; however, the relationship between BMI and muscular viscoelastic properties is still unknown. Aims: The purpose of this study was to determine the correlation of BMI with muscular viscoelastic properties, and to compare these properties in a young sedentary population with normal and overweight individuals. *Methods:* A total of 172 healthy sedentary individuals (mean age, 26.00 ± 5.45 years) were categorized by sex (male and female) and BMI classification (normal (BMI, 18.50–24.99 kg/m^2^), overweight (BMI = 25.00–29.99 kg/m^2^)). Body weight was evaluated using an electronic scale, while height was measured using a standard stadiometer. BMI was calculated by dividing the weight in kilograms by the square of height in meters. The viscoelastic properties (tone, stiffness, and elasticity) of the biceps brachii (BB) and biceps femoris (BF) muscles were measured bilaterally using the MyotonPRO device at rest. *Results:* The bilateral BF tone and stiffness, right BB stiffness, and elasticity showed weak correlations with BMI in all participants. Furthermore, the bilateral BF tone and stiffness, right BB stiffness and elasticity, and left BB stiffness were weakly positively correlated with male sex. Only the right BB elasticity was weakly positively correlated with BMI in females (*p* < 0.05). No correlation with BMI was determined for other viscoelastic properties (*p* > 0.05). The overweight group showed increased bilateral BF stiffness and tone, right BB stiffness, and reduced bilateral BB elasticity compared to the normal-weight group (*p* < 0.05), while other viscoelastic properties were similar (*p* > 0.05). Greater bilateral BB tone, BF tone and stiffness, and lower BF elasticity were observed in males than in females (*p* < 0.05), but other viscoelastic properties were not significantly different (*p* < 0.05). No effect of BMI–sex interactions was found on viscoelastic properties (*p* > 0.05). *Conclusions:* The BB and BF viscoelastic properties were weakly correlated with BMI. Males showed greater muscle tone and stiffness, and lower elasticity. The overweight individuals showed increased stiffness and tone, particularly in lower extremities, and reduced elasticity in upper extremities. The effect of BMI–sex interactions on the viscoelastic properties was not clear. Higher BMI (increased mechanical load) might cause the human body to develop different muscular viscoelastic adaptations in the extremities.

## 1. Introduction

Obesity is a significant health problem with a gradually increasing global prevalence over the past 3 decades. Obesity can be defined as excessive or abnormal fat accumulation in a manner that can impair health, and it causes a predisposition to chronic diseases [1]. The cardiovascular and metabolic consequences of obesity have been studied extensively, but less attention has been paid to its impact on muscle function. Excessive body mass may have direct or indirect effects on balance, posture, physical activity, and muscle function (i.e., overall inadequate strength and power) [2].

Muscle function—especially strength—depends on the structural and material properties of the muscle. Structural changes (fiber size and pennation, cross-sectional area) are well known to affect force generation. Previous studies have found that excessive fat infiltration into muscles is associated with reduced muscle strength and power, as well as functional limitation [3]. Muscle quality is crucial for motor performance, efficiency of activities of daily living (ADL), and control of joints. Obese people need higher absolute forces to move and support their bodies during ADL, such as climbing stairs, sitting, and walking. This can lead to abnormal loading to joints and gait mechanics, and cause malalignment, especially in the lower limb joints.

However, it is still unclear how fatty infiltration of muscle affects muscle mechanics. Healthy muscle contains approximately 2% fat (intra- and extramyocellular), which can increase up to 5% in obese people. The intramuscular fat may reach higher values as a result of hypertrophy, and change fiber types due to adaptation of mechanical loading in obese individuals. However, excessive weight gain, adipocyte hypertrophy, and fatty infiltration result in an increase in fibrous components (a decrease in contractile elements), and the reduction in the size and number of muscle fibers may lead to changes in viscoelastic properties of the muscle—namely, tone, stiffness, and elasticity.

There are few studies that have investigated the effects of BMI on the viscoelastic properties of muscles [4].Various sophisticated methods and populations have been used to evaluate these properties, including elastography, ultrasonography, and force plates [5,6,7,8,9]. In a study using a force plate, obese adolescents were found to have greater gastrocnemius muscle stiffness and lower biceps brachii elasticity than their lean counterparts. Fatty infiltration of skeletal muscles in obese people may increase muscle stiffness and reduce flexibility compared to non-obese individuals, due to the limitation of range of motion and stable posture [9]. Elastography was used in another study to predict the correlation between BMI and mechanical properties, and it was concluded that BMI was weakly correlated with upper trapezius stiffness [10]. In contrast, other studies have failed to identify any relationship between viscoelastic properties of muscles and BMI using different assessment techniques, such as shear wave ultrasound elastography [11,12].

Access to such devices is not always possible, or may be limited in most clinics, because of their high purchase and maintenance costs and the requirement of technical expertise [6]. More recently, a new handheld device known as MyotonPRO (Müomeetria Ltd., Tallinn, Estonia) has been introduced. MyotonPRO offers quick, non-invasive, cost-effective and quantitative measurement of the mechanical properties of skeletal muscles [6]. Objective measurement of soft tissue viscoelastic properties provided by MyotonPRO has high test–retest reliability and repeatability [6]. Additionally, this portable, user-friendly device has shown good-to-excellent reliability of muscle stiffness measurements in healthy individuals and those with various disease states, including stroke, cerebral palsy, and paratonia [13,14,15,16,17,18]. More recently, MyotonPRO was used in a single study to identify BMI-related differences in cervical muscle stiffness and elasticity, and a weak correlation between the upper trapezius elasticity and BMI was observed, along with a moderate correlation between BMI and stiffness of the sternocleidomastoid and upper trapezius muscles [19].

Excessive body weight and its mechanical effect may change muscle viscoelasticity differently in extremities when considering the vertical load. To the best of our knowledge, upper and lower extremity muscular viscoelastic properties are not correlated with BMI. Available data from the literature do not indicate a clear association between BMI and viscoelastic properties. Therefore, the primary aim of this study was to determine the degree of any relation of BMI to viscoelastic properties of the muscle (i.e., tone, stiffness, and elasticity). The secondary aim of this study was to identify BMI- and sex-related differences in these properties in a young sedentary population (normal vs. overweight people, males vs. females). It was hypothesized that (1) greater stiffness and tone, and lower elasticity, of the biceps brachii (BB) and biceps femoris (BF) muscles would be correlated with higher BMI; (2) muscle stiffness and tone would be greater and elasticity would be lower in overweight individuals than in normal-weight individuals; (3) muscle stiffness and tone would be greater, and elasticity would be lower, in males than in females; and (4) muscle stiffness and tone would be greater, and elasticity would be lower, in overweight males and females than their normal-weight counterparts.

## 2. Materials and Methods

### 2.1. Participants

This cross-sectional study recruited healthy individuals from the local community. A total of 172 healthy young people (mean age, 26.00 ± 5.45 years; mean height, 1.69 ± 0.08 m; mean body weight, 70.88 ± 10.99 kg; 86 females (mean age, 26.59 ± 5.39 years) and 86 males (mean age, 25.41 ± 5.47 years)) in the city of Gaziantep (Turkey) were included in this study.

Sedentary young individuals with a range of BMI values (18–30 kg/m^2^) and physical activity levels of ≤300 MET minutes/week, as reflected by the International Physical Activity Questionnaire (IPAQ) scores, were included in the study [20], because physical activity or regular exercise training could affect muscle function (intramuscular component) and viscoelastic properties [8,21]. All participants were asked to complete the IPAQ (modified version) to answer questions about their activity levels in order to determine their overall level of training [22]. Based on the responses to the questionnaire, the participants were divided into 3 categories—Category 1: low, representing a sedentary life with no moderate/vigorous activity at all, Category 2: moderate, and Category 3: high, representing a recreational lifestyle achieving a minimum of at least 600 and 3000 MET min/week, respectively. The subjects were excluded if they had a systemic or metabolic disease, mental disorder, chronic medication use, a condition that might cause muscle atrophy, prior musculoskeletal surgery in the last three months, BMI greater than 30 kg/m^2^ or less than 18 kg/m^2^, or physical activity levels of ≥300 MET min/week.

Approval for the study (dated 16.12.2020, No. 2020/101) was obtained from the Ethics Committee for Non-Invasive Research Studies of Hasan Kalyoncu University, Faculty of Health Sciences. All subjects who participated in the study were informed about the nature and purpose of the study, and signed the written consent form.

### 2.2. Procedures

The physical characteristics (body weight, height, activity level) and demographic data (age and sex) of the participants were recorded prior to the test. Their physical activity levels were assessed using the International Physical Activity Questionnaire. Body weight was measured using an electronic scale (GSE 450; GSE Scale Systems, Novi, MI, USA), while height was assessed using a standard stadiometer. BMI was calculated by dividing the weight in kilograms by the square of height in meters.

Participants were divided into two subgroups according to BMI and sex: normal (BMI = 18.50–24.99 kg/m^2^) (*n* = 86), and overweight (BMI = 25.00–29.99 kg/m^2^) (*n* = 86). Of the 86 male participants, 43 (50%) were in the normal group, and 43 (50%) were in the overweight group. Among the 86 female participants, 43 (50%) were in the normal group, and 43 (50%) were in the overweight group.

The viscoelastic properties of the biceps brachii (BB) and biceps femoris (BF) muscles were evaluated bilaterally using a MyotonPRO (Müomeetria Ltd., Tallinn, Estonia) device. The participants were asked to avoid alcohol intake for at least 24 h and strenuous physical activity for at least 48 h before the test [15]. All measurements were obtained during the afternoon by the same evaluator. The measurements were performed at the ambient temperature (22–24 °C) and humidity (45–60%) in the same environment.

The MyotonPRO is known to have good-to-excellent reliability in healthy populations [13,23]; it can be used for objective diagnosis and monitoring in soft tissues in terms of validity and inter-user reliability [24,25]. The MyotonPRO device provides data on three different properties: Tone (f) indicates a passive or resting muscle state without oscillation frequency (Hz). Stiffness (N/m) indicates resistance to any contraction or external intervention [26]. Elasticity (D) is obtained as a logarithmic reduction of the natural oscillation of soft tissues. Elasticity is inversely proportional to the decrement and, therefore, an increase in the logarithmic decrement of the muscle indicates a reduction in the muscle elasticity [27]. The measurement creates a short-duration (15 ms), low-force (0.40 N) mechanical stimulation that induces damped natural oscillations of the tissues after the constant pre-stimulation (0.18 N) of the probe placed perpendicular to the muscle (3 mm in diameter), and is obtained by recording oscillations using an accelerometer [27].

**Figure 1:**
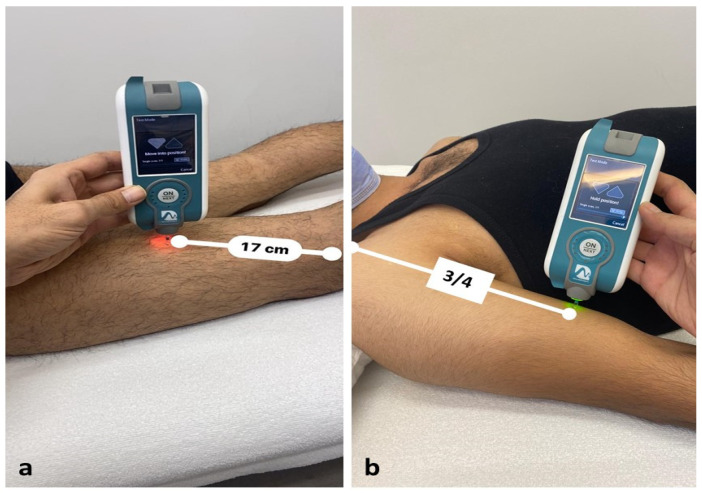
The reference points of muscles for myotonometric assessment: (**a**) biceps femoris; (**b**) biceps brachii.

Before the measurements, participants rested in supine and prone positions for 10 min and were instructed to relax as much as possible during MyotonPRO recordings on reference points of BB and BF (Figure 1). The BB mechanical properties were measured in supine position on a massage table, with the shoulder externally rotated, elbow extended, and wrist supinated. A rolled towel was placed under the wrist to flex (15°) the elbow to enable relaxation and take the stretch off the muscle. The reference point was marked with a permanent marker by palpating the lateral tip of the acromion and the mid-cubital fossa, and the device was applied at ¾ of the distance between them [28]. For the BF, the participant lay in the prone position with their feet hanging freely over the edge after placing a pillow above the ankle joint. The evaluator grasped the heel with the hip and knee in neutral position; then, the muscle was palpated while the individual performed isometric contraction against the evaluator’s hand. Along with the contraction, the most prominent part of the BF muscle was marked, and measurements were taken from this point, as recommended by Gavronski et al. [26]. These muscles were preferred since they have been studied previously in many studies, and are particularly important as they are regularly used in activities of daily living [15,29,30]. The average values obtained from three consecutive measurements from the muscle reference points were included in the statistical analysis.

### 2.3. Statistical Analysis

Descriptive statistics were presented as mean ± standard deviation. The Shapiro–Wilk test was used to check whether the data were normally distributed. The two-way analysis of variance (ANOVA) was used to assess the effects of BMI and sex. The effect size (ɳ^2^) was also reported and interpreted as small (0.2), medium (0.5), or large (0.8). The relationship between numerical variables was evaluated using Spearman’s correlation. A Spearman’s correlation coefficient of 0.00–0.10 was interpreted as a very weak correlation or no correlation, 0.10–0.39 as a weak correlation, 0.40–0.69 as a moderate correlation, 0.70–0.89 as a high correlation, and 0.90–1.00 as a very strong correlation [31]. The intraclass correlation coefficient (ICC_2,1_) was used for intra-observer reliability, using a two-way random effects absolute agreement model. The standard error of measurement (SEM_95_) and the minimal detectable change (MDC) were calculated using the following formulae: SEM95 = SD × √(1 − ICC_2,1_), and MDC = 1.96 × √2 × SEM_95_. The reliability level of ICCs was determined as follows: poor (0.00–0.20), fair (0.21–0.40), moderate (0.41–0.60), good (0.61–0.80), and excellent (0.81–1.00) [32].

Statistical analysis was conducted using SPSS for Windows (IBM Corp. Armonk, NY, USA), and a *p*-value < 0.05 was considered statistically significant. The minimum number of participants required for each group was determined to be 44 (α = 0.01) in order to detect a significant difference between two different subgroups at the large effect level (f = 0.75) with a power of 0.90. G-power version 3.9.1.7 was used in power analysis [9].

## 3. Results

### 3.1. Body Composition

Mean and standard deviation values of the participants by BMI classification are presented in Table 1.

### 3.2. Intra-Observer Reliability

In all participants, the within-day (three measurements) intra-rater reliability of the MyotonPRO in the BB and BF produced excellent ICCs for both extremities (ICC2_2,1_: 0.889–0.959). Excellent ICCs were also found for the BB and BF in both the normal-weight (ICC_2,1_: 0.913–0.970) and overweight (ICC_2,1_: 0.898–0.963) groups, as detailed in Table 2. The reliability of the right BF stiffness appeared to be lower than that of other viscoelastic properties in all participants and in overweight participants.

The SEM_95_ of the BB and BF muscles ranged from 0.44 to 0.64 Hz for tone, from 12.31 to 18.80 N/m for stiffness, and from 0.04 to 0.08 (log) for elasticity in all participants. The SEM_95_ of the BB and BF muscles ranged from 0.43 to 0.64 Hz for tone, from 10.57 to 14.76 N/m for stiffness, and from 0.03 to 0.07 (log) for elasticity in normal-weight participants. The SEM_95_ ranged from 0.38 to 0.72 Hz for tone, from 11.81 to 19.50 N/m for stiffness, and from 0.04 to 0.09 (log) for elasticity in overweight participants for the same muscles (Table 2).

The MDC of the BB and BF muscles ranged from 1.21. to 1.77 Hz for tone, from 34.40 to 52.12 N/m for stiffness, and from 0.13 to 0.21 (log) for elasticity in all participants. The MDC of the BB and BF muscles ranged from 1.19 to 1.78 Hz for tone, from 29.32 to 40.92 N/m for stiffness, and from 0.11 to 0.19 (log) for elasticity in normal-weight participants. The MDC ranged from 1.06 to 1.99 Hz for tone, from 32.75 to 54.06 N/m for stiffness, and from 0.12 to 0.26 (log) for elasticity in overweight participants for the same muscles (Table 2).

The SEM_95_ and MDC of the right BF stiffness in overweight participants were higher than those for the left BB and bilateral BF stiffness. Furthermore, these values were highest when compared to other groups.

### 3.3. Correlation between the BB and BF Viscoelastic Properties and BMI

#### 3.3.1. Study Sample

BMI was weakly correlated with right BF tone and stiffness (r = 0.17; *p* = 0.024, r = 0.19; *p* = 0.011). Weak positive correlations were also observed between BMI and left BF tone and stiffness (r = 0.19; *p* = 0.014, r = 0.21; *p* = 0.007). Weak positive correlations were found between the right BB stiffness/elasticity and BMI (r = 0.27; *p* = 0.001, r = 0.28; *p* = 0.001). No correlations were identified between BMI and other viscoelastic properties of the BB and BF muscles (*p* > 0.05) (Table 3).

#### 3.3.2. Females

BMI showed a weak positive correlation with the right BB elasticity (r = 0.25; *p* = 0.022). BMI was not correlated with other viscoelastic properties of the BB and BF muscles (*p* > 0.05) (Table 3).

#### 3.3.3. Males

Weak positive correlations were observed between right BF tone/stiffness and BMI (r = 0.22; *p* = 0.042, r = 0.26; *p* = 0.014). Weak positive correlations were also observed between left BF tone/stiffness and BMI (r = 0.27; *p* = 0.013, r = 0.30; *p* = 0.005). Weak positive correlations were found between the right BB stiffness/elasticity and BMI (r = 0.37; *p* = 0.001, r = 0.35; *p* = 0.001). The left BB stiffness showed a weak positive correlation with BMI (r = 0.22; *p* = 0.045). No correlations were found between BMI and other viscoelastic properties of the BB and BF muscles (*p* > 0.05) (Table 3).

### 3.4. Comparison of BB Viscoelastic

Right BB tone revealed a major effect of sex (*p* = 0.001, ɳ^2^ = 0.59, F(1.168) = 10.505), but no significant effects of BMI (*p* = 0.680, ɳ^2^ = 0.001) or sex × BMI (*p* = 0.150, ɳ^2^ = 0.012). Males showed greater muscle tone than females. Right BB stiffness revealed a major effect of BMI (*p* = 0.002, ɳ^2^ = 0.054, F(1.168) = 9.592), but no significant effects of sex (*p* = 0.213, ɳ^2^ = 0.009) or sex × BMI (*p* = 0.173, ɳ^2^ = 0.011). Overweight people showed higher muscle stiffness than their normal-weight counterparts. Right BB elasticity revealed a major effect of BMI (*p* = 0.001, ɳ^2^ = 0.069, F(1.168) = 12.453), without any significant effects of sex (*p* = 0.078, ɳ^2^ = 0.018) or sex × BMI (*p* = 0.671, ɳ^2^ = 0.001). Overweight subjects showed greater muscle elasticity than their normal-weight counterparts (Table 4).

Left BB tone showed a major effect of sex (*p* = 0.018, ɳ^2^ = 0.033, F(1.168) = 5.684), but no significant effects of BMI (*p* = 0.266, ɳ^2^ = 0.007) or sex × BMI (*p* = 0.665, ɳ^2^ = 0.001). Males were found to have greater muscle tone than females. Left BB stiffness showed no significant effects of BMI (*p* = 0.310, ɳ^2^ = 0.006), sex (*p* = 0.990, ɳ^2^ = 0.000), or sex × BMI (*p* = 0.470, ɳ^2^ = 0.003). Left BB elasticity showed a major effect of BMI (*p* = 0.019, ɳ^2^ = 0.032, F(1.168) = 5.604), with no significant effects of sex (*p* = 0.253, ɳ^2^ = 0.008) or sex × BMI (*p* = 0.390, ɳ^2^ = 0.004). Overweight subjects showed greater muscle elasticity than their normal-weight counterparts (Table 4).

### 3.5. Comparison of BF Viscoelastic Properties

Right BF tone showed major effects of sex (*p* = 0.000, ɳ^2^ = 0.217, F(1.168) = 46.546) and BMI (*p* = 0.033, ɳ^2^ = 0.027, F(1.168) = 4.596), but no significant sex × BMI interaction (*p* = 0.415, ɳ^2^ = 0.004). Greater muscle tone was found in males than in females, and in overweight people than in their normal-weight counterparts. Right BF stiffness showed major effects of sex (*p* = 0.000, ɳ^2^ = 0.176, F(1.168) = 35.848) and BMI (*p* = 0.015, ɳ^2^ = 0.035, F(1.168) = 6.060), but no significant sex × BMI interaction (*p* = 0.199, ɳ^2^ = 0.010). Greater muscle stiffness was found in males than in females, and in overweight people than in their normal-weight counterparts. Right BF elasticity showed a major effect of sex (*p* = 0.000, ɳ^2^ = 0.148, F(1.168) = 29.148), but no significant effects of BMI (*p* = 0.715, ɳ^2^ = 0.001) or sex × BMI (*p* = 0.800, ɳ^2^ = 0.000). Males were found to have greater muscle elasticity than females (Table 4).

Left BF tone showed major effects of sex (*p* = 0.000, ɳ^2^ = 0.126, F(1.168) = 35.848) and BMI (*p* = 0.021, ɳ^2^ = 0.031, F(1.168) = 6.060), but no significant sex × BMI interaction (*p* = 0.390, ɳ^2^ = 0.004). Greater muscle tone was detected in males than in females, and in overweight people than in their normal-weight counterparts. Left BF stiffness demonstrated major effects of sex (*p* = 0.000, ɳ^2^ = 0.146, F(1.168) = 24.126) and BMI (*p* = 0.003, ɳ^2^ = 0.050, F(1.168) = 5.441), but no significant sex × BMI interaction (*p* = 0.186, ɳ^2^ = 0.010). Greater muscle stiffness was found in males than in females, and in overweight people than in their normal-weight counterparts. Left BF elasticity showed a major effect of sex (*p* = 0.000, ɳ^2^ = 0.190, F(1.168) = 39.327), but no significant effects of BMI (*p* = 0.580, ɳ^2^ = 0.002) or sex × BMI (*p* = 0.187, ɳ^2^ = 0.010). Males were found to have greater muscle elasticity than females (Table 4).

## 4. Discussion

This study was conducted to determine the correlation of BMI with the viscoelastic properties of the BB and BF muscles, and to identify BMI- and sex-related differences in these properties in a young sedentary population (normal vs. overweight people, male vs. female). The hypotheses of this study were that (1) greater stiffness and tone, and lower elasticity, of the BB and BF muscles would be correlated with higher BMI; (2) muscle stiffness and tone would be greater, and elasticity would be lower, in overweight individuals than in normal-weight individuals; (3) muscle stiffness and tone would be greater, and elasticity would be lower, in males than in females; and (4) muscle stiffness and tone would be greater, and elasticity would be lower, in overweight males and females than in their normal-weight counterparts.

The bilateral BF tone and stiffness, as well as right BB stiffness and elasticity, showed weak positive correlations with BMI in all participants. Moreover, bilateral BF tone and stiffness, right BB stiffness and elasticity, and left BB stiffness were positively correlated with male sex, whereas only the right BB elasticity was weakly positively correlated with female sex. Kocur et al. explored the relationship between the sternocleidomastoid muscle stiffness/elasticity and BMI in females, and reported that BMI was strongly correlated with elasticity and moderately correlated with stiffness [19]. However, it should be noted that Kocur et al. studied different muscles in females only, as well as an older age range in comparison to our results. In another recent study, the relationships between gastrocnemius muscle stiffness, BMI, and other factors (e.g., age, race, anxiety) were investigated in healthy individuals 18–50 years of age. No association was found between BMI and gastrocnemius muscle stiffness at rest or during contraction [33]. In a study comparing the viscoelastic properties of erector spinae muscle in patients with ankylosing spondylitis versus healthy controls, a weak correlation was found between BMI and viscoelastic properties in patients with ankylosing spondylitis. In that study, while a moderate correlation was observed between BMI and erector spinae muscle stiffness, tone, and elasticity in male patients, female patients and healthy controls (19 females, 4 males) did not show this correlation [34]. One study examining the effects of previous hamstring injury on the sense of vibration in 16 professional soccer players did not find a correlation of BMI with muscle stiffness [35]. It was difficult for us to compare our findings with those reported by other studies due to the differences in sample size, populations, and muscles studied.

We consider that the differences between female and male subjects as observed in the present study may be attributed to physiological, physical, and hormonal differences between the sexes, and when we compared the study groups by sex, most of the viscoelastic properties of the BB and BF muscles were more favorable in males. Accordingly, for the BF muscle, greater tone and stiffness and lower elasticity were observed bilaterally in males than in females. Moreover, the bilateral BB tone was greater in males compared to females, but bilateral BB stiffness and elasticity were not significantly different. We think that the BB and BF viscoelastic properties are affected by sex to a greater extent in males than females. Our results are consistent with those of other studies that reported higher gastrocnemius stiffness and lower elasticity in males than in females [36,37].

Moreover, it has been demonstrated that males have stiffer rectus femoris and gastrocnemius muscles [28,38] However, other researchers found similar stiffness of the medial gastrocnemius and biceps brachii muscles in both sexes [28,39]. Other studies have also reported greater stiffness of the soleus and biceps brachii muscles in females than in males [40,41]. The aforementioned data suggest that there is no consensus about the effect of sex on muscle stiffness, and this may be related to the different methodological approaches used in these studies. The differences in the devices used to assess stiffness, regional muscles, and the populations studied might have caused discrepancies in the results reported.

It is well known that there are differences between the sexes in force generation, muscle fiber type, energy requirements and endurance, contraction rate, and neural activations. Force generation is dependent on internal and external factors. Regarding internal factors, muscle mass and volume, cross-sectional area, fiber type and length, and pennation angle affect strength positively, whereas changes in the muscle—including intramuscular fatty infiltration and fibrotic formations—may have a negative effect on muscle strength [42,43,44,45]. Muscle volume and mass are closely related, and both factors have a strong impact on stiffness [46,47]. It has been stated that males with greater muscle mass have increased stiffness, and a variance of 62–84% can be attributed to muscle volume [47]. Similarly, it was observed that males with greater passive hamstring stiffness have greater muscle mass [46]. Having greater muscle mass and formation of more cross-bridges contribute to force generation, and may mean more connective tissue, which increases passive stiffness [44,48]. Body mass characteristics differ between males and females, given that men have more lean mass and less fat mass than women at the same BMI [49]. We think that sex-related differences in muscle architecture and body composition may affect BF stiffness, tone, and elasticity bilaterally. However, this does not suffice to explain the absence of changes in BB stiffness and elasticity. Thus, we consider that viscoelastic properties may be affected by sex and differences in regional soft tissues—and specifically, adipose tissue distribution [50].

The adipose compartments (subcutaneous and visceral adipose) were also sex-dependent. Women tend to have a peripheral fat distribution, whereas men are prone to having a central fat distribution, and have more visceral and less subcutaneous adipose tissue [51]. It is known that women have more fat tissue accumulation in the thighs and hips [49,52]. The excessive adipose tissue is tolerated by the expansible panniculus (by extracellular matrix, collagen, and elastin), which is resisted by subcutaneous septa. The panniculus adiposus, also known as the hypodermal fat layer, stores two-thirds of the body’s total adipose tissue. The panniculus adiposus consists of mostly white adipose tissue, and is divided into two sections: the subcutaneous adipose tissue (SAT), and deep adipose tissue (DAT). The SAT involves large, almost circular fat lobules that are organized in single or multiple layers based on fat content, whereas the DAT contains more ovular fat lobules that are formed by less organized and more obliquely arranged septa. The septa are attached to the surface of the panniculus adiposus, anchoring the skin to deeper structures, wherein they are architecturally designed as a foam-like structure. With these general characteristics, the panniculus adiposus shows some differences regionally and between the sexes. The SAT is thicker in the lower extremity than in the upper extremity, and on the posterior aspect than on the anterior. The DAT tends to be thicker posterolaterally at the level of the flanks, and thinner in the anterior part of the trunk. The architectural design of the septa is mainly oriented perpendicular to the skin in females, and in a criss-cross pattern in males. Females have more fatty tissue in the SAT, and fat lobules are organized in multiple layers in certain regions, such as the breast, arm, back, and thigh. Incidentally, this septal design is among the causes of the development of cellulitis. The cited sex-related differences in the panniculus adiposus (women storing more fat in the thighs and having more SAT and fat lobules, and the panniculus being thicker in the lower extremity) support the greater BF viscoelasticity of males compared to females, and may explain similar BB stiffness and elasticity as determined in the current study. During myotonometric assessment, the panniculus adiposus on the dorsal thigh may have masked the viscoelastic properties of the BF muscle in female subjects; therefore, this should be taken into account in future studies when evaluating viscoelastic features. Males have greater muscle stiffness and tone than females, and women have greater muscle elasticity [50]. When the effect sizes were analyzed, sex showed a moderate effect on the right BF and BB tone in male subjects, but the effect of sex on other viscoelastic properties was insignificant. Generally speaking, muscle tone and stiffness may be affected by several factors, as discussed above. However, the difference in asymmetric tone may have resulted from the inability of the subjects—especially the males—to relax sufficiently within the specified period of time. As mentioned in the study’s limitations, this would have been demonstrated if we had used EMG. The dominant extremity could be another factor. However, the effect of the dominant extremity on viscoelastic properties is still debated in the literature, and no definitive conclusion has been reached about its impact.

Another reason for the greater tone and stiffness in males than in females might be the higher estrogen levels in females, which are related to collagen synthesis [53]. Estrogen and muscle stiffness are inversely related, and the beneficial effects of estrogen on viscoelastic properties has been demonstrated previously [54]. Additionally, estrogen plays an important role in fat distribution and insulin resistance, and has anti-inflammatory properties and a protective effect against obesity. The peripheral adipose distribution (thigh, hip, and subcutaneous) in females—as opposed to central (abdominal) deposition in males—has been associated with estrogen. In particular, it prevents visceral obesity, and has beneficial effects on insulin sensitivity, which is closely related to glucose homeostasis and adipose tissue metabolism [51,55]. The increase in adipokines, which regulate the production of metalloproteinases, prostanoids, and cytokines in adipose tissue, can affect skeletal muscle architecture in obesity [56]. We believe that these physiological mechanisms may lead to different adaptations in muscular and neural structures in females, and may explain why female sex was not associated with viscoelastic properties.

The overweight participants showed increased BF stiffness and tone bilaterally, increased right BB stiffness, and reduced BB elasticity bilaterally in this study. In contrast with our hypothesis, the study results showed no change in bilateral BF elasticity, bilateral BB tone, or left BB stiffness. In terms of effect sizes, although there was a statistically significant difference in the BF and BB parameters mentioned previously, the effect of BMI on viscoelastic properties was found to be small (all ɳ^2^ < 0.2). Several factors may have played a role in the different viscoelastic properties between the overweight and normal-weight subjects. The SEM_95_ and MDC values of overweight participants (except for the left BB tone and stiffness) were generally slightly greater compared to their normal-weight participants. The clinical relevance of this finding should be interpreted cautiously. Despite similar reliability (ICCs) values, standard deviation was higher in the overweight subjects (due to adipose tissue), which may have resulted in higher SEM_95_ and MDC values.

The intra-rater observer reliability produced excellent ICCs (0.901–0.959) for the measurements taken at the same session. In reliability studies using the MyotonPRO, similar results were observed for the measurements obtained from the biceps brachii (ICCs; stiffness, 0.94; tone, 0.84; elasticity, 0.78), biceps femoris (ICCs for all viscoelastic properties, 0.99), and biceps femoris (ICC; stiffness, 0.88) muscles, respectively [15,30,57]. In a separate study using the MyotonPRO with older individuals (*n* = 30), the within-day reliability was similar for tone, stiffness, and elasticity measurements of the biceps femoris muscle, with an ICC of 0.99, and SEMs of 0.09 for tone, 2.39 N/m for stiffness, and 0.02 (log) for elasticity [13]. These differential findings when compared with ours may be due to the high number of repeat measurements and the use of an elderly population in that study. In a study using the Myoton 2, it was stated that although intra-rater reliability may vary depending on the muscles, it was better than between-days intra-rater reliability [57]. High-to-excellent test reliability was obtained for measurements taken at 30 min intervals using older versions of the Myoton [58,59]. In 10 healthy 40-year-old subjects, 20 measurements taken at 1 s intervals showed an ICC of 0.80 for the right side and an ICC of 0.91 for the left side for biceps femoris stiffness [57]. We achieved excellent test reliability for the measurements taken in triplicate at 1 s intervals. Consistently, a similar finding was observed in a study of 61 stroke patients with less than Grade 2 spasticity as assessed by the MAS (Modified Ashworth Scale), which showed less variability between two measurements obtained in triplicate for the biceps brachii muscle than for other muscles (ICCs, tone 0.94, elasticity 0.93, stiffness 0.92) [59]. In that study, SEMs for tone, elasticity, and stiffness were 0.78, 0.16, 11.48, respectively, and MDCs were 1.38, 0.22, and 29.5, respectively. However, our stiffness MDC and SEM values were slightly higher. This divergence may have resulted from the difference in the populations studied; our study sample consisted of healthy young individuals, whereas the study population was middle-aged stroke patients in [59]. In addition, spasticity of stroke patients (MAS ≤ 2) might have played a role.

Variations in the reliability values obtained from different muscles have been attributed to the failure to place the Myoton probe perpendicular to the reference point, or to the differences or difficulties in positioning the individual [59,60]. Other reasons may include differential muscle geometry and the use of different reference points. Subcutaneous fat tissue thickness may result in alteration of the reference point, or may make it difficult to access. The middle part of the BB muscle body lies more medially and distally in older individuals than in younger people, and this may cause a change in the reference point calculated from the anatomic prominences or points. Furthermore, in some cases, differential viscoelastic results may be obtained—for example, in the elderly, or in those with neurological disorders. Other investigators should take into account the population or disease when interpreting their MDC and SEM values in the clinical setting.

Initially, we considered that the high BMI may act as a loading stimulus, especially in the lower extremities. The lower extremity muscle volume increases along the continuum of increasing BMI from normal to obese people [51]. The plasticity of muscles in overweight/obese individuals appears to adapt structurally, as in the case of resistance-trained muscles. Adiposity can confer a training effect similar to resistance workout, as was demonstrated previously in younger people [51]. Resting muscle tone, in the absence of neural activation, contains passive stiffness and viscoelastic properties, and may change with disease and exercise [8,21]. Accordingly, it can be argued that the mechanical loading caused by excess weight may simulate an exercise impact, improving BF tone in the lower extremity, while not affecting BB tone in the upper extremity.

On the other hand, fat is a stiffer biomaterial than muscle; higher fat infiltration into the muscle results in a stiffer base material, and this causes an increase in stiffness, which acts to resist muscle fiber shortening and transverse bulking [61]. Along with excessive weight gain, adipocyte hypertrophy, and intramuscular fatty infiltration, an increase in fibrous components (a reduction in contractile elements) and a decrease in the size and number of muscle fibers might result in diminished elasticity and increased stiffness [48,49,54]. Stiffer tissues transfer force more rapidly, but lower tissue elasticity indicates higher mechanical energy dissipated [19]. Hence, overweight or obese males have greater absolute force (low relative strength) and more fatigability than their lean counterparts [46]. This is associated with loss of muscle functionality in obese people. One reason for the loss of contractile performance is the increase in muscle stiffness as intramuscular fat content increases, which has been associated with increased adiposity, and connective tissue stiffness may lead to an increase in sarcopenic muscle [56]. Fatty infiltration of muscles may adversely affect force-generating capacity and cause pseudohypertrophy, which may also be seen in obese individuals. Nevertheless, it has been recently suggested that favorable changes occur within the muscle [48]. As such, these findings may partly explain why increased bilateral BF stiffness was found in the overweight group.

Although we did not examine intramuscular contractile or base materials in our study, the findings of increased stiffness with no changes in elasticity for the BF muscle, and similarly increased elasticity without any changes in stiffness of the BB muscle, may suggest that the aforementioned negative effects on contractile elements and fatty infiltration may not have fully occurred. In that case, excessive weight or increased BMI may have affected the stiffness and elasticity of the BB and BF muscles through a different mechanism. As such, regular physical training may affect muscular components, as can be seen in athletes, who have a larger cross-sectional area than sedentary individuals [62]. Similarly, it was reported that adaptation would occur in muscles and nerves with the increased mechanical load (training effect) caused by obesity, and as a result, young obese persons might have a larger pennation angle, cross-sectional area, and muscle thickness [63]. This finding is consistent with the findings of Tomlinson et al. [64], who found that younger obese people had 77%, 70%, and 33% larger cross-sectional areas of their gastrocnemius than underweight, normal-weight, and overweight individuals, respectively. As is known, improvement of these intramuscular elements is closely related to stiffness. However, this loading effect on the upper extremities may not reach the training threshold, while mechanical loading occurs specifically in the lower extremities during the activities of daily living, such as sitting and walking. Upper extremities lack this mechanical loading, and are not used as much as lower extremities; this may bring along a disadvantage that will result in the loss of the cross-sectional area and contractile components. It is our belief that the differential mechanic effects of increased body weight lead to an increase in BF stiffness, with no effect on BB stiffness.

In addition, during activities of daily living, obese individuals need to perform lower extremity (knees, hips) joint movements in narrow angles due to excessive stress on the articular surface. In obese individuals, the use of a narrow range of joint movements is important to generate optimal force by altering muscle/fiber length [65]. Difficulties in activities of daily living and lack of physical activity may initially reduce the use of upper extremities in overweight individuals. As it happens, lower extremities may not be affected as much as the upper extremities. The vertical loads during the activities and the intensity of closed kinetic movements may cause the elasticity to remain unchanged. In one study, it was stated that elasticity is dependent on physical activity [21]. Overweight and obese individuals may develop different adaptations to meet their different needs in their daily lives. Therefore, increased BMI can affect stability, and may provide a biomechanical advantage by changing the stiffness and tone of the lower extremity muscles due to excessive trunk oscillations in stance or walking. From a practical point of view, increased tone and muscle stiffness in relation to BMI may lead to a decrease in the risk of falls, injury, and overall muscular performance, resulting in limited ability to perform daily activities [66].

A wide range of body compositions can be seen in any BMI category, and BMI is highly correlated with body fat percentage. The calculation of BMI using height and weight in our study may represent a limitation of our study, because we did not measure subcutaneous fat tissue thickness. Therefore, it can be assumed that increased thickness of the subcutaneous fat tissue may alter the response of muscles, reduce their oscillation and frequency and, thus, affect tone [8]. In a study comparing female athletes with sedentary females, athletes were found to have lower BMI values, which was the reason for the decrease in the percentage of subcutaneous fat and the high muscle tone [21]. Thus, we considered that regional fat deposition and subcutaneous fat tissue might have altered or affected our viscoelasticity values, which should be considered as an important factor for future studies with myotonometric evaluation. However, it should be kept in mind that it is impossible to demonstrate some factors individually in an experimental setting, such as muscle fiber type and fiber size, and actual fat percentage in muscle and its distribution, which affect muscle performance in the obese population.

Additionally, we performed three consecutive measurements for each reference point, in line with other studies [6,58,67], and obtained our data using the triplescan to minimize data collection time and avoid potential variability in measurement locations and testing positions between measurements. The MyotonPRO software offers these options to select the measurement mode (triplescan or multiscan). These scan modes have an internal algorithm that compares 3 or 10 acceleration curves obtained in rapid succession. The device saves measurements from the curve closest to the arithmetic mean. Furthermore, the device shows the coefficient of variation after completing a series of measurements, in order to allow the researcher to verify whether the coefficient of variation for all parameters is less than 3%. If the coefficient of variation is higher, the measurement can be repeated under suitable test conditions.

### Limitations

Our study provides important information on the impact of BMI on the musculoskeletal system. However, a number of limitations should be noted. Firstly, we used the conventional BMI calculation, using only height and weight, with no other measures (e.g., body fat percentage, fat mass index) used as classification methods. We could have obtained more solid findings if there were participants with different body compositions.

Additionally, we did not evaluate the thickness of the panniculus adiposus and the reference points. The accumulation of excess adipose tissue into the lobules increases tensile stress on the septa, causing herniation of a single or globular cluster of papillae adiposae, which is known as skin dimpling and nodularity. This appearance of the skin results from the attachment of the connective tissue fibers to the dermis and deeper fascia, which is related to fibrosclerosis by the formation of myofibroblasts. Moreover, internal mechanical strains cause microchanges in the septa, such as disruption of the septal elastic fiber and calcification, the latter of which results in dystrophic calcification [2,7]. These factors might alter the response of muscles, reducing their oscillation and frequency, and might thus have affected the viscoelasticity values that we obtained.

Secondly, if we could evaluate whether the muscles relaxed sufficiently in the resting position using an objective method such as EMG, it would help us to better understand the viscoelastic properties of the muscle—especially the tone. Nevertheless, we believe that the findings of our study provide further insight into the relationship between BMI and the viscoelastic properties of the musculoskeletal system.

## 5. Conclusions

Several weak correlations were found between BMI and the viscoelastic properties of the BB and BF muscles. The overweight individuals showed increased stiffness and tone, particularly in the lower extremities, and reduced elasticity in the upper extremities. Males showed greater tone and stiffness, and lower elasticity, compared to females, especially in the lower extremities. The BMI–sex interaction did not appear to affect viscoelastic properties. These results suggest that higher BMI creates a loading stimulus that causes the human body to develop a different muscular viscoelastic adaptation. The increased mechanical load in the lower extremities may lead to an increase in muscle tone and stiffness. The upper extremity muscles, which lack mechanical loading, may be adversely affected. In addition to excess weight, increased tone and stiffness and decreased elasticity may produce advantageous or disadvantageous biomechanical effects on other systems and, in particular, the effects of physical activity, ambulation, and musculoskeletal functions need to be clarified.

## Figures and Tables

**Table 1:** Descriptive variables for BMI groups in both males and females.

	Normal Weight			Overweight		
	Total (*n* = 86)	Male (*n* = 43)	Female (*n* = 43)	Total (*n* = 86)	Male (*n* = 43)	Female (*n* = 43)
Age (year)	25.20 ± 4.92	24.67 ± 4.89	25.72 ± 4.96	26.80 ± 5.83	26.14 ± 5.95	27.47 ± 5.71
Body Weight (kg)	64.18 ± 9.33	69.49 ± 9.00	58.86 ± 6.13	77.58 ± 8.04	80.23 ± 7.83	74.94 ± 7.42
Height (m)	1.70 ± 0.08	1.76 ± 0.08	1.64 ± 0.53	1.68 ± 0.08	1.71 ± 0.07	1.65 ± 0.06
BMI (kg/m^2^)	22.01 ± 1.70	22.35 ± 1.65	21.67 ± 1.70	27.23 ± 1.36	27.26 ± 1.25	27.19 ± 1.47

Abbreviations—kg: kilogram; m: meter; BMI: body mass index; kg/m^2^: kilograms per square meter. Note: mean ± standard deviation was reported.

**Table 2:** Test–retest reliability results with SEM_95_ and MDC.

	All (*n* = 172)			Normal Weight (*n* = 86)			Overweight (*n* = 86)		
	ICC_2,1_ (95% CI)	SEM_95_	MDC	ICC_2,1_ (95% CI)	SEM_95_	MDC	ICC_2,1_ (95% CI)	SEM_95_	MDC
Right									
BB Tone (Hz)	0.930 (0.703–0.982)	0.56	1.56	0.937 (0.687–0.985)	0.43	1.19	0.928 (0.718–0.962)	0.67	1.85
BB Stiffness (N/m)	0.915 (0.777–0.958)	13.84	38.38	0.941 (0.824–0.963)	10.57	29.32	0.916 (0.741–0.954)	14.2	39.36
BB Elasticity (log)	0.959 (0.878–0.969)	0.04	0.13	0.970 (0.876–0.984)	0.03	0.11	0.963 (0.851–0.982)	0.04	0.12
BF Tone (Hz)	0.919 (0.548–0.957)	0.64	1.77	0.913 (0.797–0.960)	0.64	1.78	0.904 (0.762–0.951)	0.72	1.99
BF Stiffness (N/m)	0.889 (0.662–0.925)	18.80	52.12	0.925 (0.835–0.946)	14.76	40.92	0.898 (0.684–0.934)	19.5	54.06
BF Elasticity (log)	0.931 (0.740–0.968)	0.07	0.18	0.958 (0.855–0.982)	0.05	0.14	0.911 (0.805–0.966)	0.09	0.25
Left									
BB Tone (Hz)	0.938 (0.689–0.977)	0.44	1.21	0.923 (0.850–0.992)	0.51	1.42	0.944 (0.828–0.965)	0.38	1.06
BB Stiffness (N/m)	0.922 (0.766–0.948)	12.31	34.14	0.931 (0.823–0.972)	12.37	34.29	0.918 (0.799–0.943)	11.81	32.75
BB Elasticity (log)	0.901 (0.752–0.943)	0.07	0.21	0.967 (0.789–0.988)	0.04	0.11	0.928 (0.866–0.982)	0.09	0.26
BF Tone (Hz)	0.919 (0.760–0.961)	0.58	1.60	0.937 (0.794–0.975)	0.51	1.39	0.909 (0.688–0.957)	0.59	1.64
BF Stiffness (N/m)	0.931 (0.789–0.958)	13.37	37.06	0.943 (0.777–0.967)	12.04	33.39	0.918 (0.823–0.949)	13.88	38.49
BF Elasticity (log)	0.902 (0.833–0.986)	0.08	0.22	0.918 (0.704–0.970)	0.07	0.19	0.915 (0.841–0.930)	0.08	0.22

Abbreviations—ICC: intraclass correlation coefficient; CI: confidence interval; SEM: standard error of measurement; MDC: minimal detectable change; BB: biceps brachii; BF: biceps femoris; Hz: frequency; N/m: Newton/meter; log: logarithmic reduction.

**Table 3:** Correlations between the viscoelastic properties of BMI and the BB and BF muscles.

		Overall (*n* = 172)	Males (*n* = 86)	Females (*n* = 86)
Right BB Tone (Hz)	r	0.02	0.09	−0.11
Right BB Stiffness (N/m)	r	0.27 **	0.37 **	0.18
Right BB Elasticity (log)	r	0.28 **	0.35 **	0.25 *
Left BB Tone (Hz)	r	−0.06	−0.11	−0.07
Left BB Stiffness (N/m)	r	0.14	0.22 *	0.06
Left BB Elasticity (log)	r	0.13	0.16	0.12
Right BF Tone (Hz)	r	0.17 *	0.22 *	0.15
Right BF Stiffness (N/m)	r	0.19 *	0.26 *	0.16
Right BF Elasticity (log)	r	0.04	0.04	0.04
Left BF Tone (Hz)	r	0.19 *	0.27 *	0.15
Left BF Stiffness (N/m)	r	0.21 **	0.30 **	0.18
Left BF Elasticity (log)	r	−0.03	−0.17	0.02

** *p* < 0.01, * *p* < 0.05. Abbreviations—r: Spearman’s rank correlation; BB: biceps brachii; BF: biceps femoris; Hz: frequency; N/m: Newton/meter; log: logarithmic reduction.

**Table 4:** Comparison of viscoelastic properties in BMI groups.

	Normal Weight			Overweight			2 × 2 ANOVA (*p*-Values)		
Right	Total (*n* = 86)	Male (*n* = 43)	Female (*n* = 43)	Total (*n* = 86)	Male (*n* = 43)	Female (*n* = 43)	BMI Effect	Sex Effect	Interaction
BB Tone (Hz)	14.40 ± 1.72	14.68 ± 1.92	14.11 ± 1.48	14.53 ± 2.50	15.27 ± 2.98	13.79 ± 1.61	0.680	0.001 ^M^	0.150
BB Stiffness (N/m)	216.12 ± 43.55	215.70 ± 50.59	216.53 ± 35.76	237.92 ± 49.00	247.14 ± 57.19	228.70 ± 37.62	0.002 ^OW^	0.213	0.173
BB Elasticity (log)	1.00 ± 0.22	0.97 ± 0.20	1.02 ± 0.24	1.12 ± 0.24	1.08 ± 0.23	1.16 ± 0.25	0.001 ^OW^	0.078	0.671
BF Tone (Hz)	15.06 ± 2.18	15.97 ± 1.87	14.15 ± 2.10	15.71 ± 2.29	16.87 ± 2.32	14.56 ± 1.58	0.033 ^OW^	0.000 ^M^	0.415
BF Stiffness (N/m)	243.78 ± 53.90	261.95 ± 47.81	225.60 ± 54.01	262.83 ± 57.61	290.98 ± 61.07	234.67 ± 36.93	0.015 ^OW^	0.000 ^M^	0.199
BF Elasticity (log)	1.09 ± 0.25	1.20 ± 0.22	0.99 ± 0.25	1.10 ± 0.27	1.20 ± 0.31	1.01 ± 0.19	0.715	0.000 ^M^	0.800
Left									
BF Tone (Hz)	14.93 ± 2.00	15.51 ± 1.39	14.34 ± 2.33	15.60 ± 2.02	16.43 ± 1.97	14.77 ± 1.73	0.266	0.018 ^M^	0.665
BF Stiffness (N/m)	244.50 ± 50.47	258.74 ± 37.81	230.26 ± 57.53	265.52 ± 49.41	289.12 ± 48.15	241.93 ± 38.51	0.310	0.990	0.470
BF Elasticity (log)	1.14 ± 0.25	1.28 ± 0.24	1.01 ± 0.17	1.12 ± 0.26	1.21 ± 0.28	1.04 ± 0.20	0.019 ^OW^	0.253	0.390
BB Tone (Hz)	14.49 ± 1.85	14.86 ± 1.98	14.11 ± 1.66	14.19 ± 1.67	14.45 ± 1.63	13.93 ± 1.69	0.021 ^OW^	0.000 ^M^	0.390
BB Stiffness (N/m)	223.20 ± 47.10	220.79 ± 51.14	225.60 ± 43.16	230.07 ± 40.89	232.56 ± 41.27	227.58 ± 40.84	0.003 ^OW^	0.000 ^M^	0.186
BB Elasticity (log)	1.02 ± 0.23	0.98 ± 0.23	1.06 ± 0.22	1.11 ± 0.26	1.10 ± 0.33	1.11 ± 0.17	0.580	0.000 ^M^	0.187

Abbreviations—BB: biceps brachii; BF: biceps femoris; Hz: frequency; N/m: Newton/meter; log: logarithmic reduction; OW: overweight; M: male. Descriptive variables are presented as mean ± standard deviation.

## Data Availability

Not applicable.

## References

[1] Flegal K.M., Carroll M.D., Kit B.K., Ogden C.L. (2012). Prevalence of obesity and trends in the distribution of body mass index among US adults, 1999–2010. JAMA.

[2] Wang F., McDonald T., Champagne L.J., Edington D.W. (2004). Relationship of body mass index and physical activity to health care costs among employees. J. Occup. Med..

[3] Hilton T.N., Tuttle L.J., Bohnert K.L., Mueller M.J., Sinacore D.R. (2008). Excessive adipose tissue infiltration in skeletal muscle in individuals with obesity, diabetes mellitus, and peripheral neuropathy: Association with performance and function. Phys. Ther..

[4] Hamaguchi Y., Kaido T., Okumura S., Kobayashi A., Shirai H., Yagi S., Naoko K., Hideaki O., Shinji U. (2017). Impact of skeletal muscle mass index, intramuscular adipose tissue content, and visceral to subcutaneous adipose tissue area ratio on early mortality of living donor liver transplantation. Transplantation.

[5] Šarabon N., Kozinc Ž., Podrekar N. (2019). Using shear-wave elastography in skeletal muscle: A repeatability and reproducibility study on biceps femoris muscle. PLoS ONE.

[6] Feng Y., Li Y., Liu C., Zhang Z. (2018). Assessing the elastic properties of skeletal muscle and tendon using shearwave ultrasound elastography and MyotonPRO. Sci. Rep..

[7] Gapeyeva H., Vain A. (2008). Methodical Guide: Principles of Applying Myoton in Physical Medicine and Rehabilitation.

[8] Agyapong-Badu S., Warner M., Samuel D., Stokes M. (2018). Practical considerations for standardized recording of muscle mechanical properties using a myometric device: Recording site, muscle length, state of contraction and prior activity. J. Musculoskelet Res..

[9] Faria A., Gabriel R., Abrantes J., Brás R., Moreira H. (2009). Triceps-surae musculotendinous stiffness: Relative differences between obese and non-obese postmenopausal women. Clin. Biomech..

[10] Kuo W.H., Jian D.W., Wang T.G., Wang Y.C. (2013). Neck muscle stiffness quantified by sonoelastography is correlated with body mass index and chronic neck pain symptoms. Ultrasound Med. Biol..

[11] Seo A., Lee J.H., Kusaka Y. (2003). Estimation of trunk muscle parameters for a biomechanical model by age, height and weight. J. Occup. Health.

[12] Wood S., Pearsall D., Ross R., Reid J. (1996). Trunk muscle parameters determined from MRI for lean to obese males. Clin. Biomech..

[13] Bailey L., Samuel D., Warner M., Stokes M. (2013). Parameters representing muscle tone, elasticity and stiffness of biceps brachii in healthy older males: Symmetry and within-session reliability using the MyotonPRO. J. Neurol. Disord..

[14] Leonard C.T., Deshner W.P., Romo J.W., Suoja E.S., Fehrer S.C., Mikhailenok E.L. (2003). Myotonometer intra-and interrater reliabilities. Arch. Phys. Med. Rehabil..

[15] Agyapong-Badu S., Aird L., Bailey L., Mooney K., Mullix J., Warner M., Samuel D., Stokes M. (2013). Interrater reliability of muscle tone, stiffness and elasticity measurements of rectus femoris and biceps brachii in healthy young and older males. Work Pap. Health Sci..

[16] Chuang L.L., Wu C.Y., Lin K.C. (2012). Reliability, validity, and responsiveness of myotonometric measurement of muscle tone, elasticity, and stiffness in patients with stroke. Arch. Phys. Med. Rehabil..

[17] Drenth H., Zuidema S.U., Krijnen W.P., Bautmans I., van der Schans C., Hobbelen H. (2018). Psychometric properties of the MyotonPRO in dementia patients with paratonia. Gerontology.

[18] Lidström Å., Ahlsten G., Hirchfeld H., Norrlin S. (2009). Intrarater and interrater reliability of myotonometer measurements of muscle tone in children. J. Child. Neurol..

[19] Kocur P., Tomczak M., Wiernicka M., Goliwąs M., Lewandowski J., Łochyński D. (2019). Relationship between age, BMI, head posture and superficial neck muscle stiffness and elasticity in adult women. Sci. Rep..

[20] Saris W., Blair S., Van Baak M., Eaton S., Davies p Di Pietro L., Fogelholm M., Rissanen A., Schoeller D., Swinburn B., Tremblay A. (2003). How much physical activity is enough to prevent unhealthy weight gain? Outcome of the IASO 1st Stock Conference and consensus statement. Obes. Rev..

[21] Gervasi M., Sisti D., Amatori S., Andreazza M., Benelli P., Sestili P., Rocchi M.B.L., Calavalle A.R. (2017). Muscular viscoelastic characteristics of athletes participating in the European Master Indoor Athletics Championship. Eur. J. Appl. Physiol..

[22] Saglam M., Arikan H., Savci S., Inal I.D., Bosnak G.M., Karabulut E., Tokgozoglu L. (2010). International physical activity questionnaire: Reliability and validity of the Turkish version. Percept. Mot. Skills..

[23] Zinder S.M., Padua D.A. (2011). Reliability, validity, and precision of a handheld myometer for assessing in vivo muscle stiffness. J. Sport Rehabil..

[24] Schneebeli A., Falla D., Clijsen R., Barbero M. (2020). Myotonometry for the evaluation of Achilles tendon mechanical properties: A reliability and construct validity study. BMJ Open Sport Exerc. Med..

[25] Liu C.L., Li Y.P., Wang X.Q., Zhang Z.J. (2018). Quantifying the stiffness of Achilles tendon: Intra-and inter-operator reliability and the effect of ankle joint motion. Med. Sci. Monit. Med. Sci. Mon. Int. Med. J. Exp. Clin. Res..

[26] Gavronski G., Veraksitš A., Vasar E., Maaroos J. (2007). Evaluation of viscoelastic parameters of the skeletal muscles in junior triathletes. Physiol. Meas..

[27] Myoton https://www.myoton.com/technology/.

[28] Agyapong-Badu S., Warner M., Samuel D., Stokes M. (2016). Measurement of ageing effects on muscle tone and mechanical properties of rectus femoris and biceps brachii in healthy males and females using a novel hand-held myometric device. Arch. Gerontol. Geriatr..

[29] Rihvk I., Clough A., Clough P. (2010). Investigation to compare static stretching and proprioceptive neuromuscular facilitation contract–relax stretching effects on the visco-elastic parameters of the biceps femoris muscle. Int. Musculoskelet. Med..

[30] Lee Y., Kim M., Lee H. (2021). The measurement of stiffness for major muscles with shear wave elastography and myoton: A quantitative analysis study. Diagnostics.

[31] Schober P., Boer C., Schwarte L.A. (2018). Correlation coefficients: Appropriate use and interpretation. Anesth. Analg..

[32] Altman D.G. (1990). Practical Statistics for Medical Research.

[33] Hoffman L.R., Koppenhaver S.L., MacDonald C.W., Herrera J.M., Streuli J., Visco Z.L., Wildermuth N., Albin S.R. (2021). Normative Parameters of Gastrocnemius Muscle Stiffness and Associations with Patient Characteristics and Function. Int. J. Sports Phys. Ther..

[34] White A., Abbott H., Masi A.T., Henderson J., Nair K. (2018). Biomechanical properties of low back myofascial tissue in younger adult ankylosing spondylitis patients and matched healthy control subjects. Clin. Biomech..

[35] Kawai T., Takamoto K., Bito I. (2021). Previous hamstring muscle strain injury alters passive tissue stiffness and vibration sense. J. Bodyw. Mov. Ther..

[36] Blackburn J.T., Padua D.A., Weinhold P.S., Guskiewicz K.M. (2006). Comparison of triceps surae structural stiffness and material modulus across sex. Clin. Biomech..

[37] Deng L., Zhang X., Xiao S., Wang B., Fu W. (2021). Gender Difference in Architectural and Mechanical Properties of Medial Gastrocnemius–Achilles Tendon Unit In Vivo. Life.

[38] Morse C.I. (2011). Gender differences in the passive stiffness of the human gastrocnemius muscle during stretch. Eur. J. Appl. Physiol..

[39] Chino K., Takahashi H. (2018). Association of gastrocnemius muscle stiffness with passive ankle joint stiffness and sex-related difference in the joint stiffness. J. Appl. Biomech..

[40] Saeki J., Ikezoe T., Yoshimi S., Nakamura M., Ichihashi N. (2019). Menstrual cycle variation and gender difference in muscle stiffness of triceps surae. Clin. Biomech..

[41] Eby S.F., Cloud B.A., Brandenburg J.E., Giambini H., Song P., Chen S., LeBrasseur N.K., An K.-N. (2015). Shear wave elastography of passive skeletal muscle stiffness: Influences of sex and age throughout adulthood. Clinl. Biomech..

[42] Miller A.E.J., MacDougall J., Tarnopolsky M., Sale D. (1993). Gender differences in strength and muscle fiber characteristics. Eur. J. Appl. Physiol..

[43] Staron R.S., Hagerman F.C., Hikida R.S., Murray T.F., Hostler D.P., Crill M.T., Ragg K.E., Toma K. (2000). Fiber type composition of the vastus lateralis muscle of young men and women. J. Histochem. Cytochem..

[44] Shorten M.R. (1987). Muscle elasticity and human performance. Med. Sport Sci..

[45] Abe T., Brechue W.F., Fujita S., Brown J.B. (1998). Gender differences in FFM accumulation and architectural characteristics of muscle. Med. Sci. Sports Exerc..

[46] Gajdosik R., Giuliani C., Bohannon R. (1990). Passive compliance and length of the hamstring muscles of healthy men anc women. Clin. Biomech..

[47] Chleboun G.S., Howell J.N., Conatser R.R., Giesey J.J. (1997). The relationship between elbow flexor volume and angular stiffness at the elbow. Clin. Biomech..

[48] Komi P.V. (1984). Physiological and biomechanical correlates of muscle function: Effects of muscle structure and stretch—Shortening cycle on force and speed. Exerc. Sport Sci. Rev..

[49] Garaulet M., Perez-Llamas F., Fuente T., Zamora S., Tebar F.J. (2000). Anthropometric, computed tomography and fat cell data in an obese population: Relationship with insulin, leptin, tumor necrosis factor-alpha, sex hormone-binding globulin and sex hormones. Eur. J. Endocrinol..

[50] Fröhlich-Zwahlen A., Casartelli N., Item-Glatthorn J., Maffiuletti N. (2014). Validity of resting myotonometric assessment of lower extremity muscles in chronic stroke patients with limited hypertonia: A preliminary study. J. Electromyogr. Kinesiol..

[51] Geer E.B., Shen W. (2009). Gender differences in insulin resistance, body composition, and energy balance. Gend. Med..

[52] Kvist H., Chowdhury B., Grangård U., Tylen U., Sjöström L. (1988). Total and visceral adipose-tissue volumes derived from measurements with computed tomography in adult men and women: Predictive equations. Am. J. Clin. Nutr..

[53] Bell D.R., Blackburn J.T., Ondrak K.S., Hackney A.C., Hudson J.D., Norcross M.F., Padua D.A. (2011). The effects of oral contraceptive use on muscle stiffness across the menstrual cycle. Clin. J. Sport Med..

[54] Bell D.R., Blackburn J.T., Norcorss M.F., Ondrak K.S., Hudson J.D., Hackney A., Padua D.A. (2012). Estrogen and muscle stiffness have a negative relationship in females. Knee Surg. Sports Traumatol. Arthrosc..

[55] Maffiuletti N.A., Ratel S., Sartorio A., Martin V. (2013). The impact of obesity on in vivo human skeletal muscle function. Curr. Obes. Rep..

[56] Brady A.O., Straight C., Schmidt M., Evans E. (2014). Impact of body mass index on the relationship between muscle quality and physical function in older women. J. Nutr. Health Aging..

[57] Mullix J., Warner M., Stokes M. (2012). Testing muscle tone and mechanical properties of rectus femoris and biceps femoris using a novel hand held MyotonPRO device: Relative ratios and reliability. Work. Pap. Health Sci..

[58] Chuang L.L., Wu C.Y., Lin K.C., Lur S.Y. (2012). Quantitative mechanical properties of the relaxed biceps and triceps brachii muscles in patients with subacute stroke: A reliability study of the myoton-3 myometer. Stroke Res. Treat..

[59] Chuang L.L., Lin K.C., Wu C.Y., Chang C.W., Chen H.C., Yin H.P., Wang L. (2013). Relative and absolute reliabilities of the myotonometric measurements of hemiparetic arms in patients with stroke. Arch. Phys. Med. Rehabil..

[60] Bizzini M., Mannion A.F. (2003). Reliability of a new, hand-held device for assessing skeletal muscle stiffness. Clin. Biomech..

[61] Rahemi H., Nigam N., Wakeling J.M. (2015). The effect of intramuscular fat on skeletal muscle mechanics: Implications for the elderly and obese. J. R. Soc. Interface..

[62] Ryu M., Jo J., Lee Y., Chung Y.S., Kim K.M., Baek W.C. (2013). Association of physical activity with sarcopenia and sarcopenic obesity in community-dwelling older adults: The Fourth Korea National Health and Nutrition Examination Survey. Age Ageing.

[63] Garcia-Vicencio S., Coudeyre E., Kluka V., Cardenoux C., Jegu A., Fourot A.V., Ratel S., Martin V. (2016). The bigger, the stronger? Insights from muscle architecture and nervous characteristics in obese adolescent girls. Int. J. Obesity..

[64] Tomlinson D.J., Erskine R., Winwood K., Morse C., Onambélé G. (2014). The impact of obesity on skeletal muscle architecture in untrained young vs. old women. J. Anat..

[65] Maffiuletti N.A., Jubeau M., Agosti F., De Col A., Sartorio A. (2008). Quadriceps muscle function characteristics in severely obese and nonobese adolescents. Eur. J. Appl. Physiol..

[66] Barbat-Artigas S., Pion C.H., Leduc-Gaudet J.P., Rolland Y., Aubertin-Leheudre M. (2014). Exploring the role of muscle mass, obesity, and age in the relationship between muscle quality and physical function. J. Am. Med. Dir. Assoc..

[67] Pruyn E.C., Watsford M.L., Murphy A.J. (2016). Validity and reliability of three methods of stiffness assessment. J. Sport Health Sci..

